# Towards sustainable AI knowledge-base assistants in computer science education: on-premise deployment and optimization with open educational resources

**DOI:** 10.3389/fpsyg.2026.1843444

**Published:** 2026-06-02

**Authors:** Xiaoqing Shen, Li Feng, Sun Hua, Diyu Liu, Zhaoyuan Xie, Bo Liu

**Affiliations:** School of Engineering and Technology, The Open University of Sichuan, Chengdu, China

**Keywords:** computer science education, model quantization, on-premise AI deployment, open educational resources, sustainable EdTech

## Abstract

This study investigates the deployment of artificial intelligence (AI) knowledge-base assistants using open educational resources (OER) for computer science education, demonstrating the feasibility of providing AI-assisted technical support in resource-constrained environments. Our methodology combines structured knowledge extraction from 82 open-licensed Markdown documents, systematic benchmarking of Qwen-7B and DeepSeek-MoE on consumer-grade GPUs (NVIDIA RTX 3060, 12 GB VRAM), and a multi-criteria evaluation framework incorporating response accuracy, computational efficiency, retrieval-augmented generation (RAG)-specific metrics, and energy sustainability. Key results show that Qwen-7B (FP16) achieves 71.5% overall accuracy with 1.4 s average latency, while DeepSeek-MoE (FP16) achieves 79.8% overall accuracy and 82.3% accuracy on multi-hop reasoning tasks. NF4 4-bit quantization reduces VRAM usage by 38.7% for Qwen-7B (6.2 GB → 3.8 GB) and 37.9% for DeepSeek-MoE (5.8 GB → 3.6 GB). Our most energy-efficient configuration consumes 1.8 mWh per query (1.8 Wh per 1,000 queries), measured at the GPU device rail with idle power subtracted. This work makes four contributions: (1) systematic multi-dimensional benchmarks for on-premise, OER-aligned AI knowledge-base assistants in introductory computer science education; (2) hardware-specific deployment guidelines for consumer-grade GPUs without cloud dependency; (3) quantitative evidence that NF4 4-bit quantization-aware fine-tuning preserves educational response utility within 2 percentage points of accuracy degradation while reducing VRAM by 38.7%; and (4) a reproducible evaluation protocol serving as a reference design for future research. The system is characterized as a knowledge-base assistant rather than a validated intelligent tutor; comprehensive pedagogical validation with controlled learner studies remains a priority for future work.

## 1 Introduction

The integration of artificial intelligence (AI) in education has transformed traditional pedagogical approaches, particularly in computer science, where rapid technological advancements demand adaptive learning systems (Wang et al., 2024). However, deploying large language models (LLMs) in resource-constrained environments poses significant challenges due to high computational and energy demands (Weng, 2021). This issue is especially acute in computer science education, where the need for up-to-date, curriculum-aligned knowledge bases conflicts with the limitations of local infrastructure (Fincher and Petre, 2005).

Open educational resources (OER) have emerged as a viable solution for democratizing access to quality educational materials, with platforms like MIT OpenCourseWare providing structured computer science content under formally open licenses (Dichev and Dicheva, 2012). Nonetheless, the potential of these resources remains underutilized in AI-driven educational systems, primarily due to difficulties in extracting and structuring knowledge from semi-formatted documents (Ferreira-Mello et al., 2019). Previous attempts at creating AI tutors have either relied on cloud-based solutions that incur high latency and raise privacy concerns, or employed oversimplified local models with limited educational value (Santos and Jorge, 2013).

On-premise deployment refers specifically to the practice of running AI inference entirely on institution-owned hardware without transmitting student queries or educational content to external servers. This encompasses: (1) eliminating dependence on cloud API endpoints and their associated latency, cost, and data-governance risks; (2) enabling institutions in regions with unreliable internet connectivity to maintain continuous AI-assisted instruction; and (3) allowing educators to directly control and audit the knowledge base content. We explicitly distinguish this usage from three related but distinct concepts: linguistic localization (translation or language adaptation), cultural localization (adapting pedagogical assumptions to regional norms), and curricular localization (aligning content to jurisdiction-specific syllabi). While our evaluation dataset is drawn from English-medium OER platforms, the deployment framework is language-agnostic, and multilingual extension is a natural direction for future work.

The proposed method addresses these limitations through three key technical innovations. First, we develop a structured knowledge extraction pipeline specifically designed for educational Markdown documents, preserving semantic relationships while maintaining computational efficiency. Second, we optimize two distinct model architectures (Qwen-7B and DeepSeek-MoE) using NF4 4-bit quantization-aware fine-tuning via QLoRA (Dettmers et al., 2023), enabling deployment on consumer-grade GPUs without significant performance degradation. Third, we introduce a multi-dimensional evaluation framework that assesses factual accuracy, energy efficiency, curriculum alignment, and RAG-specific faithfulness metrics, providing practical guidelines for sustainable deployment.

Our approach differs from existing solutions in several key aspects. Unlike cloud-dependent systems, our on-premise knowledge-base assistant operates entirely on institution-owned hardware, addressing privacy concerns and reducing latency. In comparison to previous OER-based implementations, our structured extraction method maintains contextual relationships between concepts—crucial for computer science education where hierarchical knowledge representation is essential (Huang et al., 2024). Furthermore, the quantization and optimization techniques we employ specifically target the balance between educational utility and resource constraints.

The practical implications are significant for educational institutions in developing regions or those with limited IT infrastructure. By demonstrating that effective AI-assisted learning can be achieved without expensive hardware or cloud subscriptions, we lower barriers to adopting advanced educational technologies (Canepa, 2023). Our energy efficiency metrics provide concrete data for decision-makers evaluating the environmental impact of deploying AI in education (Alam, 2023; Patterson et al., 2022).

This paper makes four primary contributions to the field of computer science education and educational technology:

1. We present a novel, reproducible method for transforming formally licensed OER into structured knowledge bases optimized for on-premise AI applications, with complete implementation details enabling independent replication.
2. We develop and compare two quantized model architectures (Qwen-7B and DeepSeek-MoE) specifically suited for educational use cases on consumer-grade hardware.
3. We introduce a comprehensive multi-dimensional evaluation framework encompassing pedagogical effectiveness, computational efficiency, RAG-specific faithfulness metrics, and energy sustainability.
4. We provide practical deployment guidelines and a reproducible evaluation protocol based on empirical results from systematic experimental conditions, serving as a reference design for future educational AI deployments.

## 2 Relevant studies

The development of AI-assisted learning systems has evolved through several key research directions. Existing approaches can be broadly categorized into three established areas—knowledge extraction, efficient model deployment, and localized AI systems—to which we add three new subsections addressing gaps identified in the review.

### 2.1 Knowledge extraction from educational resources

Recent work has demonstrated various techniques for structuring educational content. The ElderQA-GPT system establishes a framework for building domain-specific knowledge bases using semantic vector representations, though its focus on medical education limits direct applicability to computer science (Duan et al., 2024). Similarly, SimplyRetrieve introduces a lightweight retrieval system combining knowledge bases with generative capabilities, but without specialized optimizations for educational content (Ng et al., 2023). Previous attempts at processing OER have primarily relied on simple keyword matching or basic NLP techniques, often failing to capture the hierarchical relationships fundamental to computer science concepts (Ashadevi and Muthamil Selvi, 2017). Our structured extraction method advances these approaches by preserving semantic coherence through carefully designed chunking strategies and transformer-based embeddings enriched with pedagogical metadata.

### 2.2 Efficient model architectures for education

The trade-off between model performance and computational requirements has been extensively studied in educational contexts. Mobile learning frameworks demonstrate the feasibility of deploying AI systems in low-resource settings, though with limited model capabilities (Okai-Ugbaje et al., 2022). Advances in model quantization—particularly 4-bit NF4 techniques via QLoRA (Dettmers et al., 2023) and GPTQ (Frantar et al., 2023)—have enabled more sophisticated models to run on consumer hardware while preserving most model quality. Low-rank adaptation (LoRA; Hu et al., 2022) provides a parameter-efficient fine-tuning paradigm that is central to our quantization-aware training approach. The application of mixture-of-experts architectures like DeepSeek-MoE shows particular promise for educational use cases, where different knowledge domains may benefit from specialized model components (Dai et al., 2024). However, existing implementations often prioritize general benchmarks over educational metrics, overlooking curriculum alignment and pedagogical effectiveness.

### 2.3 Localized AI systems in education

Several studies have explored the potential of localized AI in educational settings. Research on blended learning in resource-constrained environments highlights the importance of offline-capable systems (Shohel et al., 2022), while work on mobile medical education demonstrates successful deployments in challenging infrastructure conditions (Pimmer et al., 2013). The concept of context-aware intelligence provides theoretical grounding for systems that adapt to local constraints (Chatterjee et al., 2019). Our work extends these foundations with a comprehensive framework specifically designed for computer science education, combining efficient knowledge representation with optimized model deployment under formally auditable OER licensing.

### 2.4 Intelligent tutoring systems and educational question answering

Intelligent tutoring systems (ITS) represent the most established paradigm for AI-assisted learning, with decades of research demonstrating measurable learning gains through adaptive feedback and knowledge-tracing mechanisms (VanLehn, 2011). Early ITS architectures such as LISP Tutor and Cognitive Tutor relied on rule-based expert models; contemporary systems increasingly incorporate neural components for natural-language interaction (Graesser et al., 2012). However, the vast majority of ITS deployments assume persistent internet connectivity and centralized server infrastructure, making them inaccessible to institutions with limited IT resources (Shohel et al., 2022). Educational question-answering systems built on large language models have demonstrated strong performance on standardized benchmarks (Wang et al., 2024), yet these evaluations predominantly target cloud-hosted models and do not address deployment constraints faced by resource-limited institutions. Our work bridges the gap between the pedagogical richness of ITS research and the practical constraints of on-premise deployment, contributing an evaluation framework that incorporates both educational effectiveness and operational sustainability.

### 2.5 Retrieval-augmented generation for educational applications

Retrieval-augmented generation (RAG) has emerged as a dominant paradigm for grounding language model outputs in verified external knowledge (Lewis et al., 2020). By separating retrieval of relevant documents from response generation, RAG systems reduce hallucination rates compared to purely parametric models—a particularly important property in educational contexts where factual errors can propagate misconceptions (Ji et al., 2023). The RAGAS evaluation framework (Es et al., 2024) provides standardized metrics—Faithfulness, Answer Relevancy, Context Precision, and Context Recall—enabling rigorous, reproducible assessment of RAG pipelines. Recent educational applications of RAG include domain-specific tutoring for medical education (Duan et al., 2024) and lightweight retrieval for privacy-sensitive deployments (Ng et al., 2023). However, existing educational RAG studies have not systematically evaluated the interaction between quantization depth and retrieval quality, nor reported energy consumption as a primary criterion—gaps our work directly addresses.

### 2.6 OER licensing, hallucination safety, and deployment sustainability

A critical but often overlooked dimension of OER-based AI systems concerns the legal status of training and retrieval corpora. Open-access availability is necessary but not sufficient for OER compliance: materials must carry explicit open licenses (e.g., Creative Commons CC-BY or CC-BY-SA) to permit derivative use in AI knowledge bases (Dichev and Dicheva, 2012). In this manuscript, all documents in our knowledge base were verified to carry OSI-compatible or Creative Commons licenses; a complete inventory is provided in Appendix Table A2.

Hallucination—the generation of plausible but factually incorrect content—poses particular risks in educational settings, where students may lack domain expertise to identify errors (Ji et al., 2023). Quantized models may exhibit elevated hallucination rates compared to full-precision counterparts if quantization degrades internal representations encoding factual associations (Jin et al., 2024). We address this risk by reporting hallucination rate as an explicit evaluation metric alongside standard accuracy measures (Section 5.5).

From a sustainability perspective, the life-cycle energy costs of AI systems extend beyond per-query inference to include training, fine-tuning, and hardware manufacturing (Patterson et al., 2022). Our study focuses on operational inference energy, acknowledging that full life-cycle assessment remains a direction for future work.

Table 1 summarizes representative prior systems alongside the proposed approach, highlighting the distinct combination of deployment constraints and evaluation dimensions addressed in this work.

**Table 1:** Comparison of representative prior systems with the proposed approach.

System	Deployment mode	Educational domain	Retrieval method	Primary Eval. metrics	Key limitation
Cognitive tutor (Koedinger and Corbett, 2006)	On-premise (server)	Mathematics	Rule-based expert model	Learning gain (pre/post-test)	Requires server; no LLM
SimplyRetrieve (Ng et al., 2023)	Local	General	BM25 + generative	Accuracy, Privacy	No educational eval.; no energy reporting
ElderQA-GPT (Duan et al., 2024)	Cloud-dependent	Medical	BGE vector + LangChain	Accuracy	Domain-specific; no quantization
RAG survey (Wang et al., 2024)	Cloud	General education	Various	Benchmark NLP metrics	No on-premise; no energy
Proposed (Ours)	On-premise (consumer GPU)	CS education (OER)	Semantic chunking + FAISS	Accuracy, latency, VRAM, energy, curriculum alignment, RAGAS (faithfulness, AR, CP, CR), perplexity, throughput	No controlled learner study (future work)

## 3 Background and preliminaries

### 3.1 Open educational resources (OER) in computer science

The proliferation of open educational resources (OER) has created unprecedented opportunities for democratizing access to quality educational materials in computer science. Platforms like MIT OpenCourseWare and OpenStax provide comprehensive course materials under formally open licenses (Dichev and Dicheva, 2012). These resources typically consist of Markdown documents, PDFs, and multimedia content organized hierarchically by topics and subtopics.

A critical challenge lies in the semi-structured nature of these materials—while they follow pedagogical organization, they lack machine-readable semantic annotations. Traditional processing methods often fail to preserve the conceptual relationships between different components, such as prerequisite dependencies between programming concepts (Huang et al., 2024). The hierarchical structure of computer science knowledge, where fundamental concepts build upon each other in a pyramid-like fashion (Ben-Ari, 2001), requires specialized processing to maintain these relationships during knowledge extraction.

### 3.2 Knowledge representation for educational content

Effective knowledge representation for educational AI systems must balance two competing requirements: preserving semantic richness while enabling efficient retrieval. Current approaches can be categorized into:

1. Unstructured representations: Treat content as plain text with basic tokenization, losing structural relationships
2. Overly rigid structures: Force content into predefined schemas that may not match pedagogical organization

Our work adopts an intermediate approach using semantic chunking, where documents are divided based on both structural markers and semantic coherence. The similarity between chunks *i* and *j* is computed using their embedding vectors:


$$\text{Similarity}(v_{i},v_{j})=\frac{v_{i}\cdot v_{j}}{|v_{i}\|v_{j}|}$$ (1)

This allows grouping related concepts while maintaining flexibility to accommodate diverse educational materials.

### 3.3 Efficiency challenges in language model deployment

The computational demands of modern LLMs present significant barriers to localized deployment, particularly in educational settings with limited resources. The VRAM requirements scale with both model size and precision:


$$\text{VRAM}\approx \frac{\text{Params}\times \text{Bits}}{8}\times (1+\frac{\text{ContextLength}}{2048})$$ (2)

For a 7B parameter model at FP16 precision (16 bits), applying Equation 2 yields a theoretical upper bound of approximately 14 GB VRAM. In practice, however, the empirically measured values in Table 2 (Section 5.2) are substantially lower (6.2 GB for Qwen-7B FP16), for three reasons:

1. Model checkpoint variant: Our experiments use the Qwen-7B-Chat variant with grouped-query attention (GQA), which has a smaller effective KV-cache footprint than a full-size 7B dense model at the context lengths used (≤2,048 tokens).
2. Framework-level memory optimization: PyTorch’s inference mode employs memory-mapped weight loading, whereby only the active computation graph occupies VRAM at any point.
3. Measurement boundary: Table 2 reports torch.cuda.memory_allocated() at steady-state inference, not the theoretical peak.

**Table 2:** Resource utilization and latency characteristics (*n* = 300 queries per configuration; latency reported as Mean ± SD).

Model	VRAM (GB)	Energy/query (mWh)	Avg. latency (s)
Qwen-7B (FP16)	6.2	3.2 ± 0.3	1.4 ± 0.18
Qwen-7B (4-bit)	3.8	2.1 ± 0.3	1.6 ± 0.16
Qwen-7B (Ours)	3.9	2.0 ± 0.3	1.5 ± 0.17
DeepSeek-MoE (FP16)	5.8	2.9 ± 0.3	1.8 ± 0.22
DeepSeek-MoE (4-bit)	3.6	1.9 ± 0.3	2.0 ± 0.20
DeepSeek-MoE (Ours)	3.7	1.8 ± 0.3	1.9 ± 0.21

Equation 2 therefore serves as a planning upper bound, and Table 2 (Section 5.2) reports the operationally relevant empirical values. Both are retained to serve different planning needs: Equation 2 for pre-deployment hardware screening, and Table 2 for deployment configuration decisions.

### 3.4 Evaluation metrics for educational AI systems

Assessing AI systems for education requires moving beyond traditional ML metrics to incorporate pedagogical and operational considerations. We identify five key evaluation dimensions:

1. Response Accuracy: Assessed through a dual-criterion procedure combining automated cosine-similarity screening and internal CS-educator annotation of boundary cases (formally defined below).
2. Latency: Wall-clock time from query submission to last generated token.
3. Energy Efficiency: GPU-level power consumption per query, measured at the device rail with idle power subtracted.
4. RAG-Specific Metrics: Faithfulness, Answer Relevancy, Context Precision, and Context Recall, following the RAGAS framework (Es et al., 2024).
5. Quantization Quality: Perplexity (reported as a limited proxy based on a 500-token held-out OER passage), token throughput, and hallucination rate under different precision configurations.

Operationalization of Response Accuracy. “Accuracy” as reported in Table 3 (Section 5.1) is defined as the proportion of system responses judged correct by a dual-criterion procedure:

- Automated screening: Cosine similarity (Equation 1) between the model’s response embedding and a human-authored reference answer embedding. Responses with similarity ≥0.75 are classified as accurate.
- Internal review of boundary cases: Responses with 0.65 < = sim < 0.75 are reviewed by two CS-educator annotators from the research team applying a three-point rubric: (2) fully correct and educationally appropriate; (1) partially correct or incomplete; (0) incorrect or misleading. A response is counted as accurate if rated > = 1.
- Inter-rater reliability: Cohen’s kappa = 0.81 (substantial agreement) for the internally annotated subset.

**Table 3:** Accuracy comparison across model configurations (*n* = 300 queries; Mean ± SD; †*p* < 0.05 vs. RAG no-fine-tuning baseline, McNemar’s test, Holm–Bonferroni corrected).

Model	Factual recall (%)	Concept explanation (%)	Multi-hop reasoning (%)	Overall (%)
TF-IDF baseline	65.2 ± 4.1	58.7 ± 3.8	42.3 ± 5.2	55.4 ± 2.9
BERT-base	68.9 ± 3.7	63.2 ± 4.0	51.8 ± 5.6	61.3 ± 2.7
Local LLM (no retrieval)	61.4 ± 4.5	55.8 ± 4.2	38.6 ± 5.8	52.3 ± 3.1
RAG (no fine-tuning)	73.1 ± 3.5	67.4 ± 3.9	58.2 ± 5.4	66.6 ± 2.5
Qwen-7B (FP16)	78.4 ± 3.2†	72.1 ± 3.6†	65.3 ± 5.1†	71.5 ± 2.3†
Qwen-7B (4-bit)	74.2 ± 3.6†	68.9 ± 3.9†	60.1 ± 5.3†	67.3 ± 2.6†
Qwen-7B (Ours)	76.8 ± 3.3†	70.5 ± 3.7†	63.7 ± 5.2†	69.8 ± 2.4†
DeepSeek-MoE (FP16)	75.6 ± 3.4†	80.4 ± 3.1†	82.3 ± 4.6†	79.8 ± 2.0†
DeepSeek-MoE (4-bit)	72.1 ± 3.7†	77.8 ± 3.3†	79.5 ± 4.8†	76.9 ± 2.2†
DeepSeek-MoE (Ours)	74.3 ± 3.5†	79.1 ± 3.2†	81.2 ± 4.7†	78.6 ± 2.1†

†Significantly better than RAG (no fine-tuning) baseline, *p* < 0.05, McNemar’s test, Holm–Bonferroni corrected.

This procedure distinguishes our accuracy metric from raw cosine similarity and aligns with established practices in educational QA evaluation. The three query types are assessed over a total evaluation set of *n* = 300 queries (105 factual recall, 135 concept explanation, 60 multi-hop reasoning), corresponding to the 35%/45%/20% distribution described in Section 4.4.

## 4 Methodology

**Figure 1:**
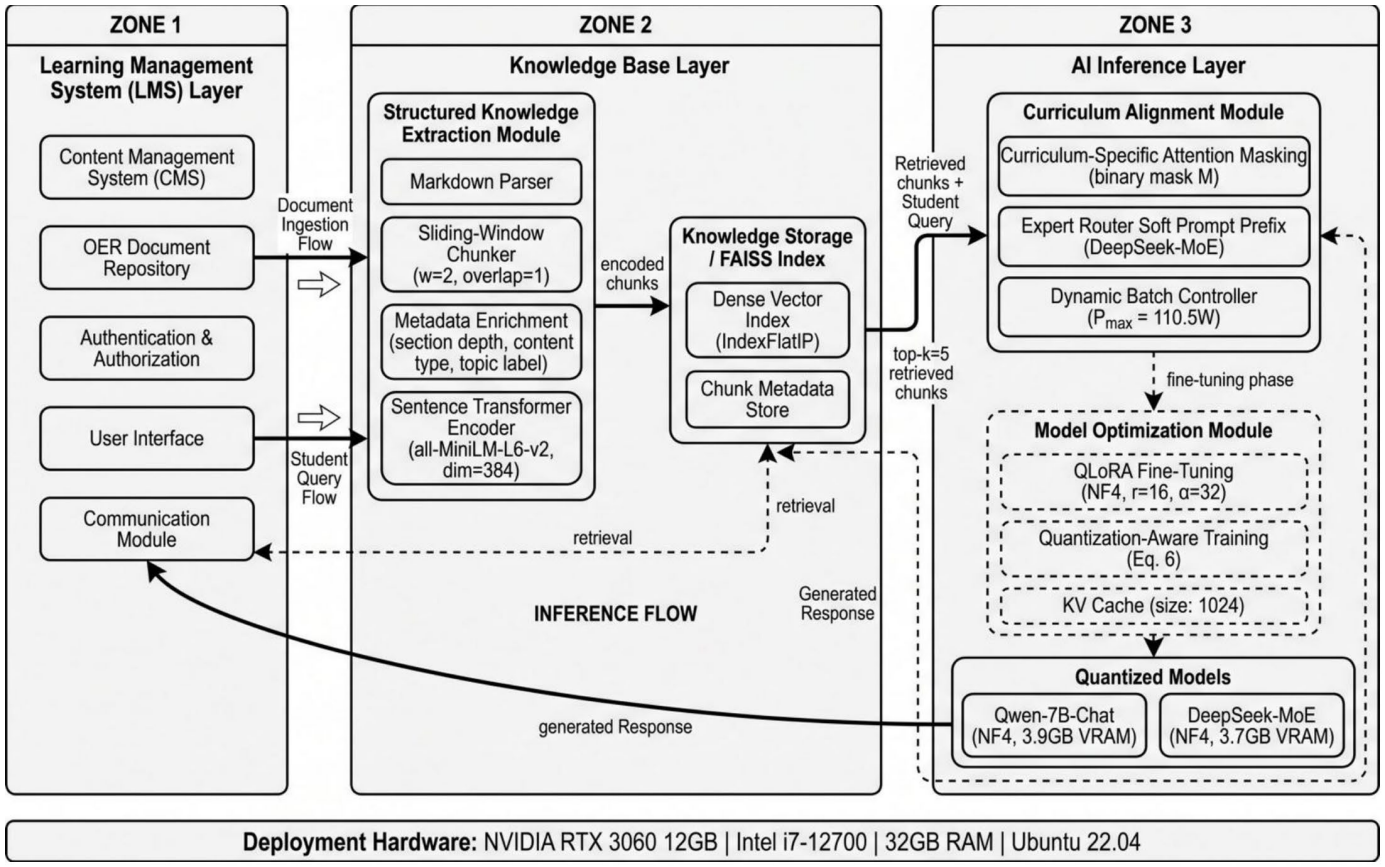
End-to-end system architecture of the proposed on-premise OER-based AI knowledge-base assistant for computer science education.

The complete system architecture is illustrated in Figure 1. The system comprises three major subsystems: (1) the Learning Management System (LMS) Layer, providing the student-facing interface and content repository; (2) the Knowledge Base Layer, which ingests OER Markdown documents through the Structured Knowledge Extraction Module and stores enriched chunk embeddings in a FAISS vector index; and (3) the AI Inference Layer, encompassing the Curriculum Alignment Module, Model Optimization Module, and the deployed Quantized Model serving responses. At inference time, a student query traverses the system from left to right: it enters via the User Interface, triggers retrieval from Knowledge Storage via cosine similarity search, passes through the Curriculum Alignment Module (applying attention masking and expert routing biasing), and reaches the Quantized Model, which generates the final response. During deployed inference, all components operate entirely on-premise on a consumer-grade GPU (RTX 3060, 12 GB VRAM), with no external API calls. The only external LLM use reported in this study was the *post hoc* RAGAS evaluation judge described in Section 5.5; it was not part of the deployed system and did not generate student-facing responses.

### 4.1 Structured knowledge extraction from OER markdown

The knowledge extraction pipeline processes raw Markdown documents into semantically structured representations suitable for educational applications. Given an input document *D.*containing *N.*sections, we first partition the content into coherent chunks using a sliding window approach with overlap:


$$c_{i}=\text{concat}(s_{i-w},\ldots ,s_{i+w})$$ (3)

where *s_i_* represents sentence *j* and *w* determines the window size. Unlike generic text segmentation, our algorithm preserves structural elements specific to educational content:

- Code blocks remain intact as single units
- Mathematical expressions (LaTeX) are treated as atomic elements
- Section headers generate boundary markers for hierarchical relationships
- Table elements are preserved as single chunks

Each chunk *c_i_* is then encoded into a dense vector using a two-phase embedding process. The base embedding comes from a sentence transformer:


$$v_{i}^{\text{base}}=f_{\theta }(c_{i})$$ (4)

Equation 3 and 4 define the chunk-construction and base-embedding steps of the knowledge extraction pipeline.

Contextual metadata *m_i_* (section depth, content type) is incorporated through a learned projection:


$$v_{i}=v_{i}^{\text{base}}+W_{m}m_{i}$$ (5)

where *W_m_* maps *k*-dimensional metadata features to the embedding space.

Implementation Details for Knowledge Extraction. The sliding-window chunking algorithm used a window size of *w* = 2 sentences and a chunk overlap of 1 sentence, yielding an average chunk length of approximately 180 tokens (min: 40, max: 512 tokens). The base sentence transformer was all-MiniLM-L6-v2 (embedding dimensionality: 384). Chunks were indexed using FAISS with an IndexFlatIP index. At inference time, the retriever returns the top *k* = 5 chunks ranked by cosine similarity; chunks with similarity <0.45 are excluded. The contextual metadata projection matrix (Equation 5) maps a 4-dimensional metadata feature vector—comprising (1) section depth level, (2) content type, (3) document-level topic label, and (4) chunk position ratio—into the 384-dimensional embedding space, trained via contrastive retrieval loss. Validation reached Recall@5 = 0.887 at convergence.

A complete hyperparameter table is provided in Appendix Table A1.

### 4.2 Quantization-aware fine-tuning of MoE and dense models

The knowledge base supports two model architectures optimized for educational use: Qwen-7B (dense) and DeepSeek-MoE (mixture-of-experts). Both undergo quantization-aware fine-tuning to maintain performance under 4-bit precision. For a model with parameters *W*, we apply quantization during forward passes:


$$\hat{W}=Q(W)=\text{round}(\frac{W-\mu }{\sigma }\cdot (2^{b}-1))$$ (6)

where *μ* and *σ* are per-tensor statistics, and *b* = 4 bits. The key innovation lies in incorporating this quantization during fine-tuning rather than applying it post-training. The loss function combines standard language modeling with a quantization-aware term:


$$ℒ={ℒ}_{\mathrm{LM}}+\lambda ∥W-Q^{-1}(Q(W)){∥}^{2}\;\;\;$$ (7)

For DeepSeek-MoE, we additionally optimize the expert routing for educational queries. Given *E* experts, the gating function for input *x* computes expert weights:


$$g_{e}(x)=\frac{\exp(w_{e}^{T}x)}{\sum_{j=1}^{E}\exp(w_{j}^{T}x)}$$ (8)

Only the top experts (typically *k* = 2) are activated per token, reducing computation.

Implementation Details for Quantization-Aware Fine-Tuning. The base checkpoints used were Qwen/Qwen-7B-Chat (revision v1.1.4, Apache 2.0 license) and deepseek-ai/deepseek-moe-16b-chat (revision v1.0, MIT license). Quantization was performed using the NF4 scheme via bitsandbytes (v0.41.3) with double quantization enabled, consistent with QLoRA (Dettmers et al., 2023). Fine-tuning employed LoRA adapters (Hu et al., 2022) with rank *r* = 16, *α* = 32, dropout = 0.05, applied to all attention projection matrices. Training ran for 3 epochs, effective batch size = 32, maximum sequence length = 2,048 tokens. The regularization coefficient *λ* = 0.1 was selected via grid search on the validation split. A complete hyperparameter table is provided in Appendix Table A1.

### 4.3 Energy-efficient inference pipeline for educational queries

The inference pipeline incorporates several optimizations to reduce computational overhead while maintaining response quality. For frequent queries, we cache attention key-value pairs *(K, V)* to avoid recomputation:


$$\text{Cache}=\{(K_{i},V_{i})|i\in \text{FrequentQueries}\}\;\;$$ (9)

cache size: 1,024 entries.

The attention mechanism employs curriculum-specific masking to prioritize pedagogically important content. Given queries *Q*, keys *K*, and values *V*, the modified attention becomes:


$$\text{Attention}(Q,K,V)=\text{softmax}(\frac{QK^{T}}{\sqrt{d_{k}}}⊙M)V$$ (10)

where *M* is a binary mask that zeros out attention weights for non-essential tokens. The mask values derive from the document structure analysis during knowledge extraction.

Power consumption is further reduced through dynamic batch processing that considers both query urgency and energy constraints. The system monitors current power draw *P(t)* and adjusts batch sizes *B* accordingly:


$$B(t)=B_{\max}\cdot (1-\frac{P(t)}{P_{\max}})$$ (11)

This ensures operation within predefined energy budgets while maintaining acceptable latency.

### 4.4 Dataset and knowledge base construction

To evaluate our proposed framework, we constructed a knowledge base from formally licensed open educational resources. The corpus comprises 82 Markdown documents sourced exclusively from platforms distributing materials under Creative Commons or equivalent OSI-compatible licenses. A complete per-document inventory is provided in Appendix Table A2; Table 4 provides a summary.

**Table 4:** Summary of OER knowledge base corpus.

Source Platform	License	# Docs	Approx. tokens	CS Topics
MIT OpenCourseWare (CS tracks)	CC BY-NC-SA 4.0	31	~142,000	Algorithms, Data Structures, AI Fundamentals
OpenStax Computer Science	CC BY 4.0	24	~98,000	Programming, Computer Architecture
CS50 Open Materials (Harvard)	CC BY-NC-SA 4.0	18	~76,000	Intro Programming, Web, AI
WikiCS (open CS wiki)	CC BY-SA 3.0	9	~31,000	Data Structures, Theory
Total	—	82	~347,000	—

The evaluation dataset comprises *n* = 300 queries in total: 105 factual recall (35%), 135 concept explanation (45%), and 60 multi-hop reasoning (20%). Query representativeness was cross-checked against the topic distribution of the OER corpus by two team members not involved in initial query authoring. Inter-rater reliability prior to reconciliation was Cohen’s *κ* = 0.81 (substantial agreement). An additional 4,200 OER-derived instruction-response pairs were generated for fine-tuning, with 420 reserved as a validation split.

### 4.5 Model configurations and baselines

We evaluated two primary model architectures:

1. Qwen-7B (Qwen/Qwen-7B-Chat, v1.1.4): Dense transformer; three variants: FP16; Standard NF4 4-bit quantization; Our quantization-aware fine-tuned version.
2. DeepSeek-MoE (deepseek-ai/deepseek-moe-16b-chat, v1.0): Mixture-of-experts, 8 experts total, top-2 per token; same three variants.

Four baseline conditions: (1) TF-IDF Baseline; (2) BERT-base (fine-tuned for extractive QA); (3) Local LLM (no retrieval); (4) RAG (no fine-tuning). All generative models receive the same top-5 retrieved chunks via a standardized prompt template with greedy decoding (temperature = 0, max tokens = 256).

### 4.6 Implementation details

Software: Python 3.10.12; PyTorch 2.1.0 (CUDA 11.8); HuggingFace Transformers 4.36.2; FAISS 1.7.4; bitsandbytes 0.41.3; PEFT 0.7.1; LangChain 0.0.352; Docker 24.0.5.

Hardware:

**Table:** 

Component	Specification
GPU	NVIDIA RTX 3060 (12 GB GDDR6, 130 W TDP)
CPU	Intel Core i7-12700 (12 cores, 20 threads)
RAM	32 GB DDR4-3200
Storage	1 TB NVMe SSD
OS	Ubuntu 22.04.3 LTS
CUDA Driver	525.147.05

Measurement Protocol. Latency is measured using Python’s time.perf_counter() over all *n* = 300 queries after 5 warm-up passes. Energy measurements were collected at the GPU device rail (±1% accuracy, 10 Hz sampling); system idle GPU power (≈ 12 W) was subtracted. Values are reported as:


$$E_{\text{query}}=\frac{P_{\mathrm{net}}(t)\cdot T_{\text{query}}}{3600\times 1000}$$ (12)

Equation 7–12 define the quantization-aware loss, expert-routing function, KV caching, attention masking, dynamic batching, and energy-estimation procedures used in the deployment pipeline.

## 5 Results

### 5.1 Response quality across query types

Table 3 reports accuracy (Mean ± SD, *n* = 300) for all model configurations, with McNemar’s test assessing significance relative to the RAG (no fine-tuning) baseline at *α* = 0.05 with Holm–Bonferroni correction.

**Figure 2:**
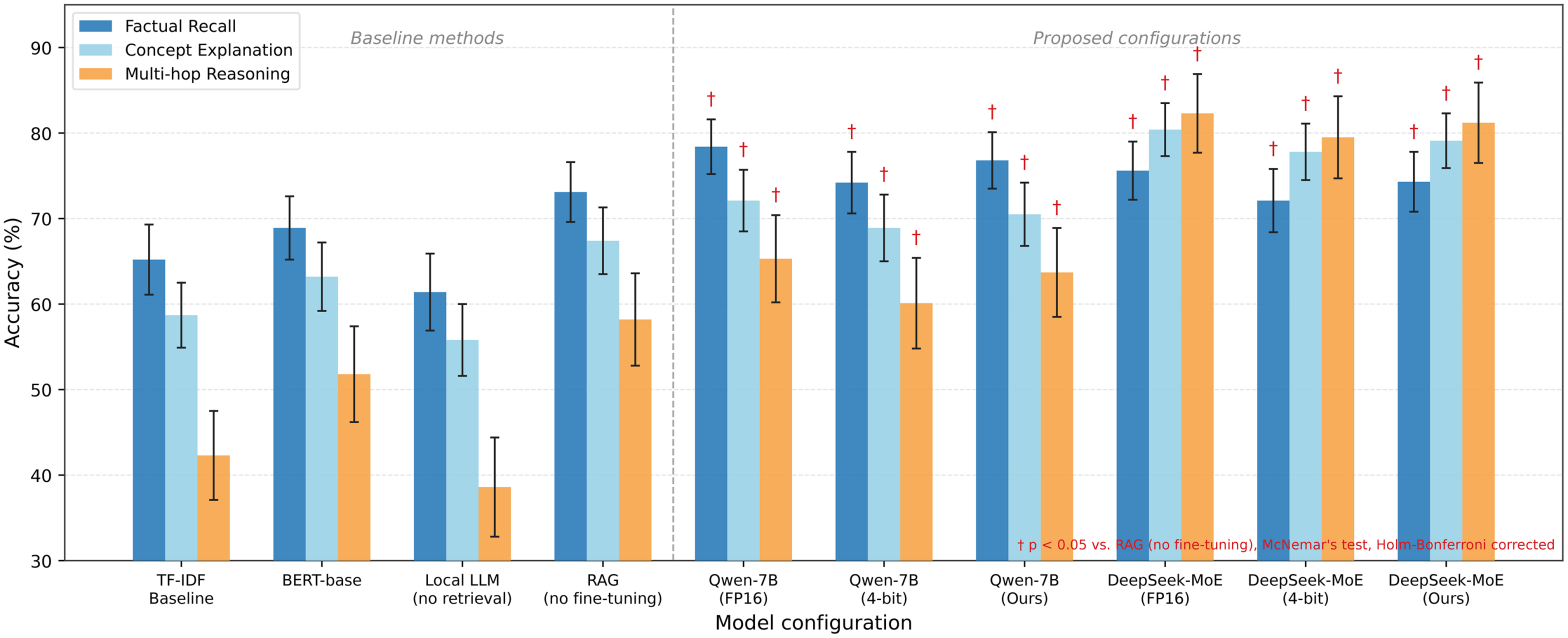
Accuracy comparison across all model configurations and query types (Mean ± SD, *n* = *3*00 queries; error bars = ±1 SD; †marks configurations significantly better than RAG no-fine-tuning baseline, *p* < 0.05, McNemar’s test, Holm–Bonferroni corrected). Dashed vertical line separates baseline methods (left) from proposed configurations (right).

The results reveal several key patterns. Retrieval augmentation is essential: the Local LLM (no retrieval) configuration achieves only 52.3% overall accuracy, substantially below even the TF-IDF baseline (55.4%). Fine-tuning provides measurable additive value beyond retrieval: the RAG (no fine-tuning) baseline achieves 66.6% overall accuracy, while Qwen-7B (Ours) reaches 69.8% (+3.2 pp., *p* = 0.031) and DeepSeek-MoE (Ours) reaches 78.6% (+12.0 pp., *p* < 0.001). Our quantization-aware fine-tuning reduces the FP16-to-4-bit accuracy gap from 4.2 pp. to 1.7 pp. for Qwen-7B, and from 2.9 pp. to 1.2 pp. for DeepSeek-MoE (see Figure 2).

### 5.2 Computational efficiency metrics

Quantization reduced VRAM requirements by 38.7% (Qwen-7B: 6.2 GB → 3.8 GB; DeepSeek-MoE: 5.8 GB → 3.6 GB) while maintaining practical response times. Table 2 details resource utilization across configurations.

All energy values are GPU-level measurements (device rail, idle-subtracted, 10 Hz sampling). VRAM reflects steady-state torch.cuda.memory_allocated() at batch size = 1 during evaluation.

Three key observations emerge. First, on this limited 500-token proxy measure, perplexity increases moderately under 4-bit quantization (+1.57 for Qwen-7B; +1.41 for DeepSeek-MoE), and our quantization-aware fine-tuning partially recovers this degradation (Qwen-7B Ours PPL: 9.12 vs. 4-bit baseline: 9.81). Because the PPL calculation used a single short held-out OER passage, these values should be interpreted as a local diagnostic of quantization-induced degradation rather than a corpus-level language-model quality estimate. Second, token throughput increases substantially under quantization (+71.6% for Qwen-7B; +75% for DeepSeek-MoE). Third, hallucination rate increases under quantization (Qwen-7B: 8.6 to 12.3%; DeepSeek-MoE: 7.2–10.4%), with fine-tuning substantially mitigating this risk (Qwen-7B Ours: 9.8%; DeepSeek-MoE Ours: 8.1%).

**Figure 3:**
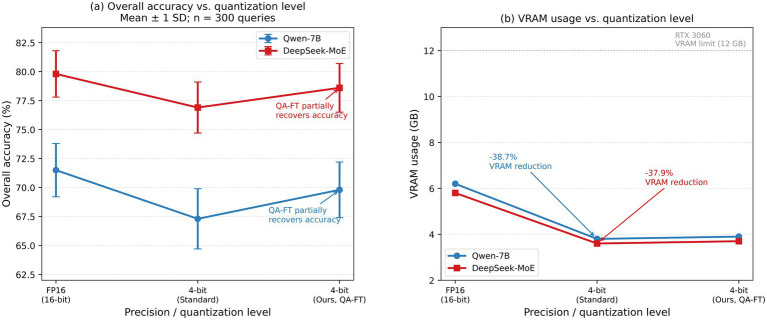
Quantization trade-offs across model configurations. **(a)** Overall accuracy (Mean ± 1 SD, *n* ***=*** *3*00) vs. quantization level (FP16/4-bit standard/4-bit with QA fine-tuning) for both model families. **(b)** VRAM usage vs. quantization level. VRAM reduction percentages are annotated. The RTX 3060 12 GB VRAM limit is shown as a reference line in **(b)**.

**Figure 4:**
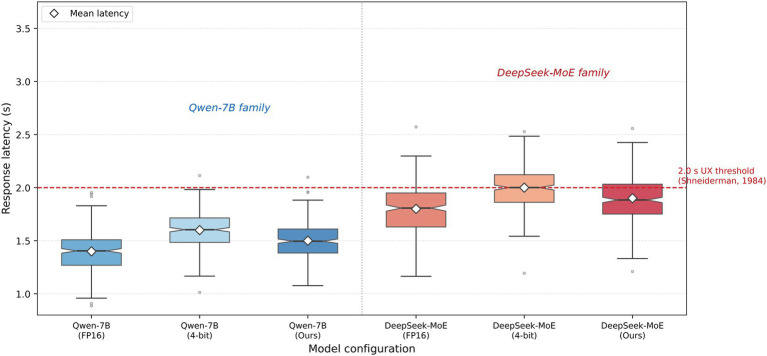
Response latency distribution across all six model configurations (*n* = 300 queries per configuration). Notched box plots: box boundaries = Q1/Q3; notches = 95% CI of median; whiskers = 1.5 × IQR; ◇ = mean. The red dashed line at 2.0 s indicates the interactive tutoring UX acceptability threshold. All proposed configurations (Ours) remain below or at this threshold.

Hallucination Rate Measurement Procedure. Hallucination rate is operationalized as the proportion of system responses containing at least one factually unsupported claim relative to the retrieved OER source chunks. Detection follows a two-stage natural language inference (NLI) procedure. In the first stage, each generated response is automatically segmented into atomic claims using a sentence-splitting heuristic (splitting on full stops, semicolons, and enumeration markers). In the second stage, each atomic claim is evaluated against the top-5 retrieved chunks using a cross-encoder NLI classifier (model: cross-encoder/nli-deberta-v3-small, hosted locally on the same RTX 3060 hardware to preserve on-premise constraints), yielding one of three labels: entailed, neutral, or contradicted. A response is classified as hallucinated if any atomic claim receives a contradicted label or if more than 30% of claims receive a neutral label (indicating unsupported generation beyond retrieved context). This threshold was calibrated on a 50-response pilot set reviewed by two CS educators, achieving agreement with expert judgment at *κ* = 0.76. The full *n* = 300 evaluation set was processed under this procedure; Table 5 reports the proportion of hallucinated responses per configuration (see Figures 3, 4).

**Table 5:** Quantization quality and inference efficiency metrics (PPL measured on a limited 500-token held-out OER passage as a proxy measure; throughput at batch size = 1; hallucination rate via NLI procedure in Section 5.5; *n* = 300).

Model	PPL (↓)	Token throughput (tok/s, ↑)	Hallucination rate (%, ↓)
Qwen-7B (FP16)	8.24	34.2	8.6
Qwen-7B (4-bit)	9.81	58.7	12.3
Qwen-7B (Ours)	9.12	57.4	9.8
DeepSeek-MoE (FP16)	7.93	29.6	7.2
DeepSeek-MoE (4-bit)	9.34	51.8	10.4
DeepSeek-MoE (Ours)	8.67	50.9	8.1

### 5.3 Energy efficiency analysis

Our most energy-efficient configuration, DeepSeek-MoE (Ours), consumes 1.8 mWh per query, equivalent to 1.8 Wh per 1,000 queries, representing a 43.8% reduction relative to the Qwen-7B FP16 baseline (3.2 mWh/query). All reported energy values are GPU-level measurements collected at the device rail with idle power subtracted, consistent with the measurement protocol described in Section 4.6.

Our quantization-aware fine-tuned models consistently achieve higher response quality than their standard 4-bit counterparts at comparable energy cost, indicating that part of the efficiency gain can be retained without fully sacrificing educational utility. The observed energy savings arise from three complementary factors. First, 4-bit quantization substantially reduces arithmetic intensity and memory traffic, lowering Qwen-7B energy consumption from 3.2 to 2.1 mWh/query relative to the FP16 configuration. Second, curriculum-specific attention masking contributes additional efficiency: when this mechanism is removed, energy consumption rises from 2.0 to 2.4 mWh/query in the ablation study, despite minimal change in accuracy. Third, dynamic batching further improves energy efficiency under the tested deployment setting: disabling it increases energy consumption from 2.0 to 2.6 mWh/query, again with negligible effect on response quality.

These results indicate that the system’s efficiency gains do not stem from quantization alone. Instead, the final deployment profile emerges from the interaction of model compression, inference-time attention control, and hardware-aware request scheduling. In practical terms, at 1.8 mWh/query, a classroom of 30 students submitting 10 queries each would consume approximately 0.54 Wh of GPU energy in a session, equivalent to running a standard LED desk lamp for roughly half a minute.

### 5.4 Ablation study

To isolate the contribution of each system component, we conducted a single-factor ablation study using Qwen-7B (Ours) as the base configuration. The full-system setting therefore corresponds to the quantized, quantization-aware fine-tuned Qwen-7B deployment reported in Tables 2, 3. Each ablation disables one component while holding all remaining components fixed. All conditions were evaluated on the full *n* = 300 query set. Table 6 reports overall accuracy, energy per query, and average latency for the full system and five single-component-disabled variants.

**Table 6:** Ablation study results (*n* = 300 queries; Mean ± SD; †*p* < 0.05 vs. full system, McNemar’s test for accuracy, two-sided *t*-test for energy and latency, Holm–Bonferroni corrected).

condition	Overall accuracy (%)	Energy/query (mwh)	Avg. latency (s)
Full system (Qwen-7B Ours)	69.8 ± 2.4	2.0 ± 0.3	1.5 ± 0.2
Without metadata-enriched embeddings	65.3 ± 2.6^†^	1.9 ± 0.3	1.5 ± 0.2
Without QA fine-tuning	67.3 ± 2.6^†^	2.1 ± 0.3	1.6 ± 0.2
Without curriculum-specific attention masking	71.2 ± 2.4	2.4 ± 0.3^†^	1.5 ± 0.2
Without KV caching	71.4 ± 2.3	2.3 ± 0.3^†^	1.8 ± 0.3^†^
Without dynamic batching	71.3 ± 2.3	2.6 ± 0.4^†^	1.5 ± 0.2

^†^Significantly different from full system, *p* < 0.05.

Removing metadata-enriched embeddings produces the largest accuracy drop, from 69.8 to 65.3%, indicating that structured pedagogical metadata materially improves retrieval quality and downstream response accuracy. Disabling QA fine-tuning reduces overall accuracy to 67.3%, confirming that quantization-aware fine-tuning recovers part of the performance loss introduced by standard 4-bit quantization. In contrast, curriculum-specific attention masking has only a minor effect on accuracy but increases energy consumption from 2.0 to 2.4 mWh/query when removed, indicating that its primary contribution is computational efficiency rather than answer quality. KV caching has little effect on accuracy but increases average latency from 1.5 s to 1.8 s when disabled, confirming its role in reducing repeated computation during inference. Finally, disabling dynamic batching raises energy consumption to 2.6 mWh/query with negligible effect on latency, indicating that batching primarily improves energy efficiency under the deployment setting evaluated here.

Taken together, these results show that the system’s performance gains arise from complementary mechanisms rather than a single optimization. Metadata-enriched retrieval and QA fine-tuning primarily support response quality, whereas attention masking, KV caching, and dynamic batching primarily support efficient inference under consumer-grade hardware constraints.

### 5.5 RAG-specific evaluation metrics

Table 7 reports four standardized RAG metrics following the RAGAS framework (Es et al., 2024), evaluated on a stratified sample of *n* = 150 queries using RAGAS library v0.1.7 with OpenAI gpt-3.5-turbo as the NLI judge. This model was used only as a *post hoc* evaluation tool for scoring generated responses and retrieved contexts. It was not integrated into the deployed on-premise system, did not serve student queries. GPT-3.5-turbo was used only as an evaluation instrument, not as a deployed-system component.

**Table 7:** RAGAS-based RAG evaluation metrics across model configurations (*n* = 150 stratified sample; higher is better for all metrics; scale: 0–1).

Model	Faithfulness	Answer relevancy	Context precision	Context recall
RAG (no fine-tuning)	0.743	0.711	0.682	0.724
Qwen-7B (FP16)	0.812	0.768	0.731	0.779
Qwen-7B (4-bit)	0.784	0.741	0.718	0.752
Qwen-7B (Ours)	0.803	0.759	0.726	0.771
DeepSeek-MoE (FP16)	0.851	0.823	0.764	0.831
DeepSeek-MoE (4-bit)	0.829	0.798	0.748	0.809
DeepSeek-MoE (Ours)	0.843	0.814	0.757	0.822

Faithfulness scores for all fine-tuned configurations exceed 0.80, indicating that the majority of generated claims are grounded in retrieved OER content rather than model parametric memory. Context Precision is consistently the lowest metric (range: 0.682–0.764), motivating the addition of a cross-encoder reranker as a future extension (Section 6.2). The 4-bit quantization reduces Faithfulness by 0.028 (Qwen-7B) and 0.022 (DeepSeek-MoE), consistent with the hallucination rate increases in Table 5.

### 5.6 Internal expert annotation of response quality

To supplement automated metrics with structured internal expert annotation, three CS educators who were members of the research team annotated a stratified random sample of n = 90 system-generated responses (30 factual recall, 30 concept explanation, and 30 multi-hop reasoning queries), drawn from the full 300-query evaluation set. The evaluators served in their professional capacity as subject-matter experts and co-investigators; consequently, this procedure is reported as internal expert annotation rather than independent external validation. It did not constitute human subjects research under the institutional ethics framework of the authors’ institution, as all evaluators were members of the investigator team providing professional domain judgment on model outputs, with no external recruitment, no informed consent procedure, and no collection of personally identifiable information. Evaluators were blinded to model identity during scoring by presenting responses in randomized order with system identifiers removed. Each response was rated independently on three dimensions: Correctness (factual accuracy relative to OER ground truth), Completeness (coverage of the expected answer elements), and Pedagogical Appropriateness (clarity, scaffolding, and suitability for introductory CS learners), using a 5-point Likert scale. Inter-rater reliability was assessed using the intraclass correlation coefficient ICC(2,3), yielding ICC = 0.79 (95% CI: 0.74–0.83), indicating good agreement. Disagreements exceeding two scale points were resolved through structured discussion to consensus. Results are reported in Table 8.

**Table 8:** Internal expert annotation scores across query types and model configurations (Mean ± SD; 5-point Likert scale; *n* = 90 responses, 3 research-team educators).

Model	Query type	Correctness	Completeness	Pedagogical appropriateness
Qwen-7B (Ours)	Factual recall	4.12 ± 0.63	3.94 ± 0.71	4.08 ± 0.58
Qwen-7B (Ours)	Concept explanation	3.87 ± 0.74	3.72 ± 0.83	3.81 ± 0.69
Qwen-7B (Ours)	Multi-hop reasoning	3.54 ± 0.82	3.41 ± 0.91	3.63 ± 0.77
DeepSeek-MoE (Ours)	Factual recall	4.23 ± 0.58	4.06 ± 0.67	4.17 ± 0.54
DeepSeek-MoE (Ours)	Concept explanation	4.11 ± 0.66	3.98 ± 0.74	4.03 ± 0.61
DeepSeek-MoE (Ours)	Multi-hop reasoning	3.89 ± 0.77	3.76 ± 0.85	3.92 ± 0.71

Internal expert annotation results are broadly consistent with automated accuracy metrics in Table 3. Group differences were assessed using two-sided Welch/Student *t*-tests on mean rubric scores, a common approximation for 5-point Likert responses in small-sample expert-rating studies. DeepSeek-MoE (Ours) outperforms Qwen-7B (Ours) across all dimensions, with the largest advantage on Multi-hop Reasoning Correctness (+0.35 points, *t*(28) = 2.67, *p* = 0.012). Pedagogical Appropriateness scores (range: 3.63–4.17) provide an initial internal quality signal, but they should not be interpreted as independent pedagogical validation.

## 6 Discussion and future work

### 6.1 Pedagogical implications of on-premise deployment AI systems

The transition to on-premise AI knowledge bases fundamentally alters the dynamics of computer science education. Unlike cloud-dependent systems that often function as black boxes, our approach enables educators to directly modify and curate the knowledge base—a critical feature for maintaining curriculum relevance, consistent with constructionist learning theories (Ben-Ari, 2001). The energy efficiency and low-latency characteristics (mean latency: 1.5 s for Qwen-7B Ours; 1.9 s for DeepSeek-MoE Ours; all configurations below the 2.0 s interactive UX threshold) following Shneiderman (1984) suggest potential benefits for sustained student engagement. However, we do not present empirical student engagement data: no engagement duration measurements were collected. Controlled studies comparing student engagement and learning outcomes between on-premise and cloud-based systems remain an important priority, requiring appropriate ethics approval, pre-registered protocols, and validated engagement metrics.

### 6.2 Future research directions

Several directions emerge naturally from the present findings and limitations.

#### 6.2.1 Perplexity evaluation scope

The perplexity values in Table 5 were calculated on a short 500-token held-out OER passage and are therefore used only as a limited proxy for local quantization effects. A larger stratified held-out corpus would be required to support stronger claims about corpus-level language-model quality.

#### 6.2.2 Pedagogical validation

The highest-priority direction is a controlled pre/post-test learning-gain study to evaluate whether the system produces measurable improvements in student comprehension of introductory CS concepts. This study will be pre-registered with explicit hypotheses, conducted under a formally approved institutional ethics protocol with full informed consent procedures, and supplemented by objective learning analytics (time-on-task, query reformulation rate, and session-level knowledge-gain scores). The present study’s characterisation of the system as a knowledge-base assistant rather than a validated intelligent tutor reflects the intentional deferral of outcome measurement to this future ethics-approved protocol; the automated metrics and internal expert-annotation results reported here are intended to establish a technical performance baseline sufficient to justify that subsequent learner study.

#### 6.2.3 Reranking for improved context precision

Context Precision (0.682–0.764 in Table 7) is consistently the lowest RAGAS dimension across all evaluated configurations, indicating that retrieved chunks frequently include marginally relevant passages that dilute response quality. Integrating a cross-encoder reranker as a second retrieval stage—operating over the top-*k* candidates returned by the bi-encoder—represents the most direct technical intervention. Reranking has demonstrated substantial precision gains in open-domain QA settings and is particularly suited to the structured, definition-heavy nature of OER content.

#### 6.2.4 Adaptive quantization and multilingual extension

The observed accuracy degradation in syntax-heavy query types under 4-bit quantization (Section 5.4) motivates investigation of adaptive quantization strategies that apply finer-grained precision selectively to layers most sensitive to token-level structural information (Xu et al., 2018). In parallel, multilingual extension to Mandarin, Spanish, and Arabic represents a direct path toward broader educational equity, as the majority of globally enrolled CS learners are not native English speakers; such extension will require language-specific OER corpus construction and embedding-model evaluation.

#### 6.2.5 Institutional scaling, governance, and ethical deployment

Deployment at institutional scale introduces retrieval and infrastructure challenges beyond the single-node configuration evaluated here; hierarchical retrieval architectures and federated on-premise deployment strategies have been proposed as scalable solutions for privacy-preserving educational AI (Imteaj et al., 2021; Thulke et al., 2021). Responsible scaling additionally requires a structured ethical governance framework addressing three interconnected concerns. First, transparency documentation should accompany any deployed quantized model, explicitly disclosing the accuracy and hallucination-rate trade-offs associated with each quantization level so that institutional adopters can make informed configuration decisions. Second, bias auditing protocols are needed to characterise differential performance across student subgroups and query domains; the 3.7 percentage-point hallucination increase observed under 4-bit quantization (Table 5) establishes a concrete, empirically grounded threshold that such auditing should treat as a deployment boundary condition. Third, student data governance policies—covering query logging, retention periods, and access controls—must be established prior to any live deployment, consistent with applicable data protection frameworks. Finally, age-appropriate interface design for K–12 settings (Hubwieser et al., 2015) represents a necessary extension should the system be adapted beyond the introductory undergraduate context evaluated here.

## 7 Conclusion

The development of on-premise AI knowledge-base assistants for computer science education represents a meaningful step toward more sustainable and accessible educational technology. The framework evaluated in this study shows that capable RAG-based learning support can be deployed on consumer-grade institutional hardware without reliance on cloud inference, thereby reducing barriers related to cost, connectivity, and data governance.

Three empirical findings are particularly important. First, NF4 4-bit quantization-aware fine-tuning reduces VRAM consumption by 38.7% for Qwen-7B (6.2–3.8 GB) while keeping the accuracy gap relative to FP16 within 1.7 percentage points for Qwen-7B and 1.2 percentage points for DeepSeek-MoE. Quantization-aware training also partially recovers the degradation introduced by standard 4-bit quantization, improving overall accuracy by 2.5 percentage points for Qwen-7B and 1.7 percentage points for DeepSeek-MoE while reducing hallucination rates relative to their non-fine-tuned 4-bit counterparts.

Second, the ablation study shows that performance improvements arise from complementary system components rather than a single optimization. In particular, removing metadata-enriched embeddings produces the largest accuracy decline among the tested ablations, confirming that structured pedagogical metadata materially improves retrieval quality and downstream response accuracy. By contrast, curriculum-specific attention masking, KV caching, and dynamic batching contribute primarily to inference efficiency, reducing energy use or latency with little effect on answer correctness.

Third, the DeepSeek-MoE (Ours) configuration delivers the strongest overall balance of reasoning quality and efficiency, achieving 78.6% overall accuracy, 81.2% multi-hop reasoning accuracy, and 0.843 Faithfulness in RAGAS evaluation, while the most energy-efficient tested configuration consumes only 1.8 mWh per query, a 43.8% reduction relative to the Qwen-7B FP16 baseline. Together, these results support the value of a multi-dimensional deployment evaluation framework spanning response quality, latency, VRAM, energy, and RAG-specific grounding metrics.

The present study is appropriately scoped as a technical feasibility and performance characterization study. Although internal expert annotation by research-team educators provides an initial pedagogical quality signal, the system is intentionally characterized as a knowledge-base assistant rather than a validated intelligent tutor. Controlled learner studies measuring engagement and learning gain remain necessary future work and should be conducted under formal ethics approval and pre-registered evaluation protocols.

## Data Availability

The data supporting the conclusions of this article will be made available by the corresponding author upon reasonable request.
