# Supplementary material for: Towards sustainable AI knowledge-base assistants in computer science education: on-premise deployment and optimization with open educational resources
Source: Front Psychol. 2026 Jun 2;17:1843444. doi: 10.3389/fpsyg.2026.1843444 (PMC13269062; doi:10.3389/fpsyg.2026.1843444)
Supplement: Supplementary file 1 [file Supplementary_file_1.docx]

Supplementary Appendix

**Article:** Towards sustainable AI knowledge-base assistants in computer science education: on-premise deployment and optimization with open educational resources

**Authors:** Xiaoqing Shen, Li Feng, Sun Hua, Diyu Liu, Zhaoyuan Xie, and Bo Liu

**Article ID:** 1843444

# Appendix Table A1. Quantization-Aware Fine-Tuning Hyperparameters

| **Hyperparameter** | **Qwen-7B** | **DeepSeek-MoE** |
| --- | --- | --- |
| Base checkpoint | Qwen-7B-Chat v1.1.4 | deepseek-moe-16b-chat v1.0 |
| License | Apache 2.0 | MIT |
| Quantization scheme | NF4 (bitsandbytes v0.41.3) | NF4 (bitsandbytes v0.41.3) |
| LoRA rank r | 16 | 16 |
| LoRA α | 32 | 32 |
| LoRA dropout | 0.05 | 0.05 |
| LoRA target modules | W_Q, W_K, W_V, W_O | W_Q, W_K, W_V, W_O |
| Learning rate (peak) | 2 × 10⁻⁴ | 2 × 10⁻⁴ |
| LR schedule | Cosine w/ warmup (ratio = 0.03) | Cosine w/ warmup (ratio = 0.03) |
| Epochs | 3 | 3 |
| Effective batch size | 32 (4 × 8 grad. accum.) | 32 (4 × 8 grad. accum.) |
| Max sequence length | 2,048 tokens | 2,048 tokens |
| λ selection (Eq. 6) | Grid search {0.01, 0.05, 0.1, 0.5}; val. PPL | Same |
| Optimal λ | 0.1 | 0.1 |
| Router modification | N/A (dense model) | Soft prompt prefix (inference only) |
| Fine-tuning samples | 4,200 (420 val. split) | 4,200 (420 val. split) |

# Appendix Table A2. OER Knowledge Base - Per-Source Inventory Summary

| **#** | **Source Platform** | **License** | **Doc Count** | **Approx. Tokens** | **Primary CS Topics** |
| --- | --- | --- | --- | --- | --- |
| 1.0 | MIT OpenCourseWare — 6.006 Introduction to Algorithms | CC BY-NC-SA 4.0 | 12 | ~58,000 | Sorting, Graph algorithms, Dynamic programming |
| 2.0 | MIT OpenCourseWare — 6.004 Computation Structures | CC BY-NC-SA 4.0 | 10 | ~47,000 | Computer architecture, Digital logic |
| 3.0 | MIT OpenCourseWare — 6.034 Artificial Intelligence | CC BY-NC-SA 4.0 | 9 | ~37,000 | Search, Machine learning fundamentals |
| 4.0 | OpenStax — Introduction to Computer Science | CC BY 4.0 | 14 | ~62,000 | Programming fundamentals, OOP |
| 5.0 | OpenStax — Principles of Computer Science | CC BY 4.0 | 10 | ~36,000 | Computer architecture, Networks |
| 6.0 | CS50 Open Materials — CS50x | CC BY-NC-SA 4.0 | 12 | ~48,000 | C, Python, Web development |
| 7.0 | CS50 Open Materials — CS50 AI | CC BY-NC-SA 4.0 | 6 | ~28,000 | Search, ML, NLP basics |
| 8.0 | WikiCS — Data Structures | CC BY-SA 3.0 | 5 | ~18,000 | Arrays, Trees, Graphs, Hash tables |
| 9.0 | WikiCS — Theory of Computation | CC BY-SA 3.0 | 4 | ~13,000 | Automata, Complexity |
|  | Total | — | 82 | ~347,000 | — |

**Note.** The full per-document inventory is available from the corresponding author upon reasonable request.
